# Targeting progastrin enhances radiosensitization of colorectal cancer cells

**DOI:** 10.18632/oncotarget.17274

**Published:** 2017-04-20

**Authors:** Aline Kowalski-Chauvel, Valerie Gouaze-Andersson, Alix Vignolle-Vidoni, Caroline Delmas, Christine Toulas, Elizabeth Cohen-Jonathan-Moyal, Catherine Seva

**Affiliations:** ^1^ Cancer Research Center of Toulouse (CRCT), UMR1037 Inserm/University Toulouse III Paul Sabatier, Toulouse, France; ^2^ IUCT Oncopole, Toulouse, France

**Keywords:** colorectal cancer, radioresistance, progastrin, cell signalling, ionizing radiations

## Abstract

A high percentage of advanced rectal cancers are resistant to radiation. Therefore, increasing the efficacy of radiotherapy by targeting factors involved in radioresistance seems to be an attractive strategy. Here we demonstrated that the pro-hormone progastrin (PG), known to be over-expressed in CRC, and recognized as a pro-oncogenic factor, is a radioresistance factor that can be targeted to sensitize resistant rectal cancers to radiations. First, we observed an increase in PG mRNA expression under irradiation. Our results also demonstrated that down-regulating PG mRNA expression using a shRNA strategy, significantly increases the sensitivity to irradiation (IR) in a clonogenic assay of different colorectal cancer cell lines. We also showed that the combination of PG gene down-regulation and IR strongly inhibits tumours progression *in vivo*. Then, we demonstrated that targeting PG gene radiosensitizes cancer cells by increasing radio-induced apoptosis shown by an increase in annexin V positive cells, caspases activation and PARP cleavage. We also observed the up-regulation of the pro-apoptotic pathway, JNK and the induction of the expression of pro-apoptotic factors such as BIM. In addition, we demonstrated in this study that inhibition of PG gene expression enhances radiation-induced DNA damage. Our data also suggest that, in addition to increase radio-induced apoptosis, targeting PG gene also leads to the inhibition of the survival pathways, AKT and ERK induced by IR.

Taken together, our results highlight the role of PG in radioresistance and provide a preclinical proof of concept that PG represents an attractive target for sensitizing resistant rectal tumours to irradiation.

## INTRODUCTION

Rectal cancers account for about 40% of total colorectal cancers (CRC). The standard treatment for advanced rectal cancers consists of a preoperative chemoradiotherapy followed by surgery. Although this approach reduces the risk of local recurrence, and increase the probability of sphincter-preserving surgery, clinical response to chemoradiotherapy varies greatly and a high percentage of advanced rectal cancers are resistant to this preoperative treatment. Only 4 to 20% of these patients achieve a pathologically complete response and 30-40% die within 5 years [1, 2]. An attractive strategy to improve the curative resection rate and to reduce the high risk of metastasis could be the increase of radiotherapy cytotoxic effect on tumours cells by specifically targeting factors involved in radioresistance [3]. However to date, there is no targeted therapy used in clinic to radiosensitize rectal tumours. Therefore there is a need for better understanding radioresistance mechanisms and to identify new factors that might be targeted to increase the response to radiotherapy.

In CRC, pre-progastrin gene is highly expressed. Indeed this gene is a target of the oncogenic pathways frequently activated in this cancer such as the APC/beta-catenin or K-ras pathways [4, 5]. However, because CRC cells lack the enzymes required to process PG into mature gastrin forms, PG is mainly produced by the cancer cells. High concentrations of PG are found in colorectal tumours and in blood of 80% of patients with colorectal cancer [6–8]. In contrast, this hormone precursor is absent from the healthy intestinal epithelium. The role of PG in cancer cells proliferation has been clearly established [9–15]. In addition in transgenic mice overexpressing PG, the risk of CRC development when treated with the carcinogen, azoxymethane is increased [16–18].

In tumours radioresistance, the potential role of PG has never been studied. In this study, we therefore tested whether the overexpression of PG observed in CRC has a functional role in mediating resistance to radiotherapy. Our data indicate that targeting PG gene expression increases radiosensitization *in vitro* and *in vivo*.

Our observations demonstrate a novel role for PG in tumours radioresistance and suggest that this pro-hormone is an attractive therapeutic target for sensitizing resistant rectal cancers to ionizing radiations.

## RESULTS

### Down-regulation of PG gene expression radiosensitizes CRC cells *in vitro*

**Figure 1:**
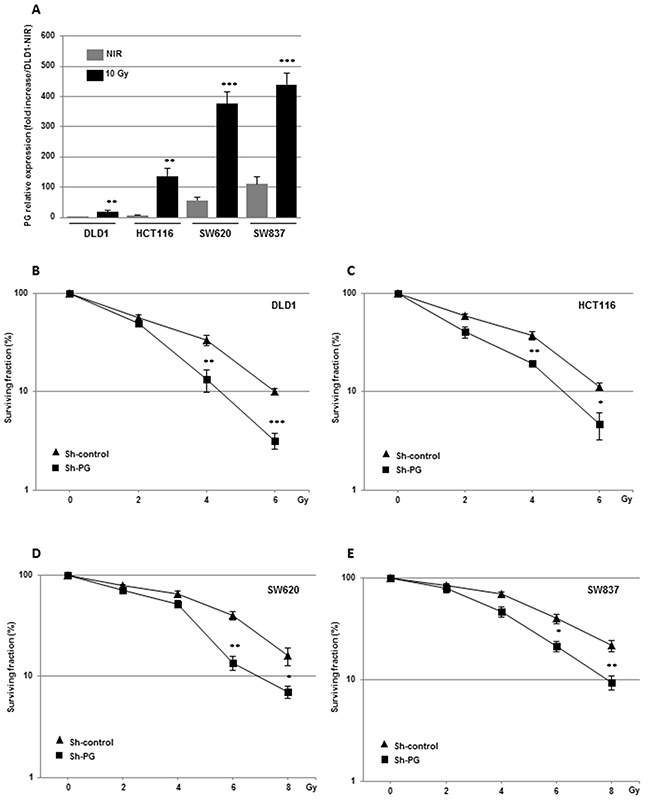
Down-regulation of PG gene expression radiosensitizes CRC cells *in vitro* **(A)** PG mRNA expression was measured (72h post-irradiation) in the different CRC cell lines following a radiation dose of 10 Gy administered as a unique dose. Clonogenic assays **(B-E)** were performed, in the different CRC cell lines stably transfected with a PG shRNA or a scramble control. Quantifications of 3 experiments are presented as means ± SD. ***P < 0.001; **0.001 < P < 0.01; *0.01 < P < 0.05.

It is well established that IR can induce pro-survival signaling pathways and the expression of factors involved in radioresistance [23]. Therefore, before evaluating if PG overexpression is functionally relevant for mediating radioresistance, we investigated whether IR could increase PG gene expression in different CRC cell lines. HCT116 and SW837 cell lines were exposed to different radiation doses administered as a unique dose (4 to 10 Gy) or by daily multifractions of 4 × 2.5 Gy. PG mRNA expression was quantified 3h to 72h post-irradiation (Supplementary Figure 1A, 1B). Although we observed in both cell lines a significant increase in PG mRNA expression with the lowest dose (4 Gy), 72h post-irradiation, the highest increase was obtained with 10 Gy (Supplementary Figure 1A). The time course revealed a significant increase 48h post-irradiation for both cell lines and a maximal effect at 72h with a radiation dose of 10 Gy (Supplementary Figure 1B). We have chosen the conditions of 10 Gy and 72h to test two other cell lines (DLD1, SW620). In every tested cell lines we observed an increase in PG mRNA expression (Figure 1A). Therefore except for the clonogenic assays we pursued the study with a unique dose of 10 Gy.

Cells were then stably transduced with a shRNA against PG previously validated in different CRC cell lines [19]. As shown in the reference [19] and Supplementary Figure 1C, 1D, both, basal PG mRNA expression or induced by irradiation were significantly inhibited by the specific PG shRNA. Inhibition of PG expression by the specific PG shRNA was also confirmed at the protein level by an immuno-fluorescent approach (Supplementary Figure 1E). Clonogenic survival assays were performed with increasing doses of radiation (Figure 1B-1E). The survival fractions normalized to the plating efficiency, were significantly decreased in all CRC cell lines expressing the PG shRNA compared to the control shRNA, indicating that down-regulation of PG mRNA expression radiosensitizes CRC cells. Conversely, addition of exogenous PG to cells with low levels of PG gene expression (HCT116) or SW837 cells in which PG gene expression has been knockdown using shRNA, increased cell survival after an irradiation dose of 6 Gy (Supplementary Figure 2).

### Targeting PG in CRC cell lines increases radio-induced apoptosis

Next we investigated the cellular mechanisms leading to the radiosensitization of cancer cells expressing the PG shRNA. These experiments were performed in HCT116 and SW837 which present different pattern of mutations (respectively p53 wild type/PTEN-PI3K mutated, p53 mutated/PTEN-PI3K wild type).

**Figure 2:**
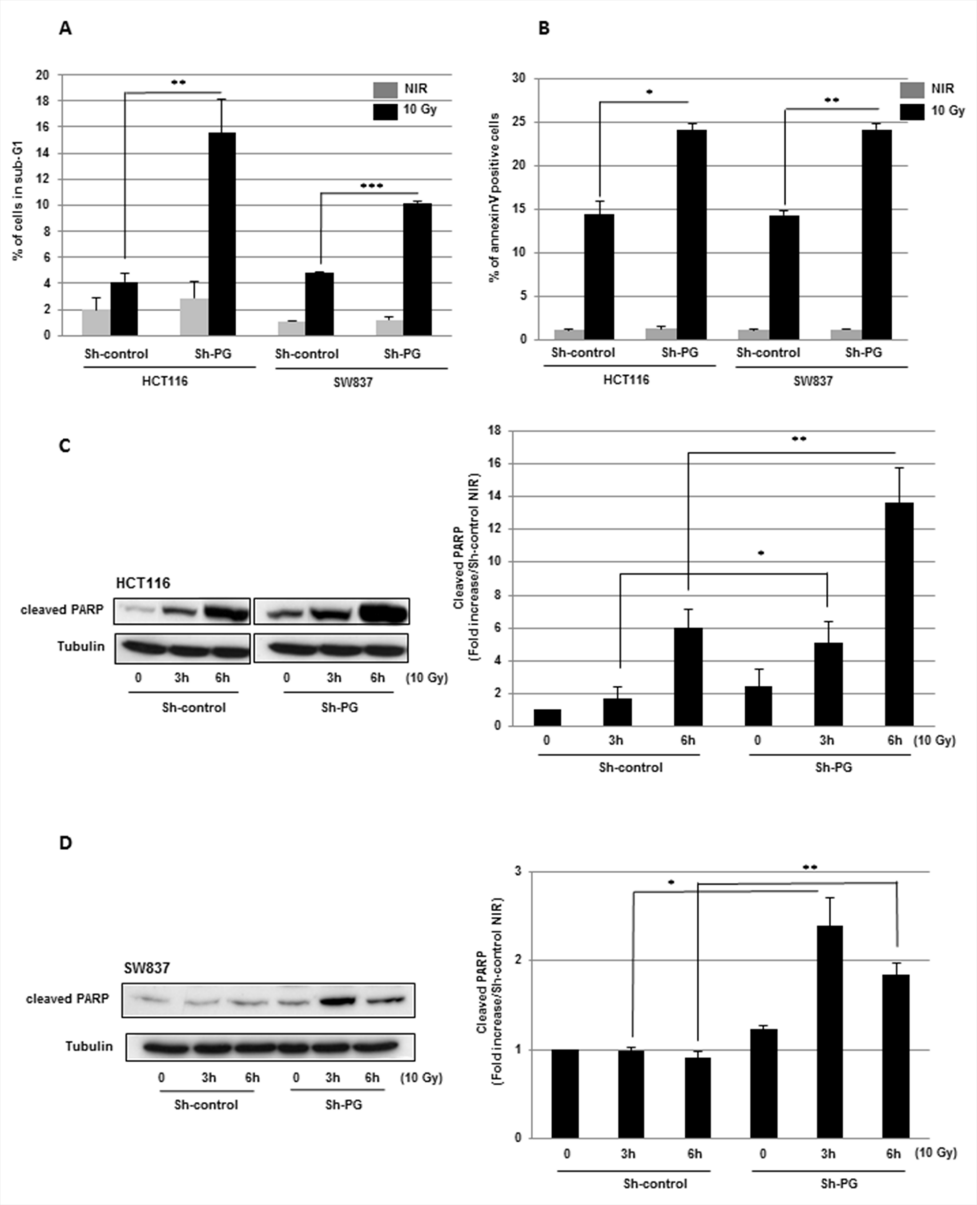
Targeting PG gene in CRC cell lines increases radio-induced apoptosis **(A)** Propidium iodide staining was performed as described in “methods” and the DNA content was analyzed by flow cytometry. Percentages sub-G1 cell population in HCT116 and SW837 are presented. **(B)** Annexin V staining was performed according to the manufacturer protocol and cells were analyzed by flow cytometry. **(C, D)** The cleavage of poly-(ADP-ribose)-polymerase-1 (PARP-1), was measured by western blot using an antibody directed against cleaved PARP-1. Quantifications of 3 experiments are presented as means ± SD. **0.001 < P < 0.01; *0.01 < P < 0.05.

First, we observed that PG gene down-regulation resulted in a significant increase of radio-induced cell death shown by the augmentation of sub-G1 fraction (Figure 2A).

**Figure 3:**
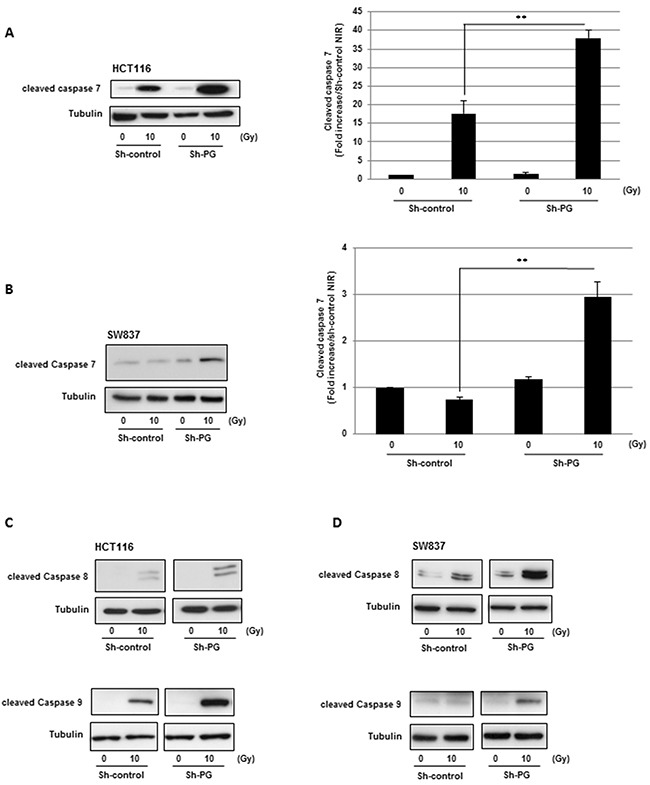
Down-regulation of PG gene expression in CRC cells increases radio-induced caspases cleavage The cleavage of caspases 7 **(A, B)**, caspases 8 and 9 **(C, D)** was measured, 6h post-irradiation, by western blot analysis using an antibody directed against the different cleaved caspases as indicated. Quantifications of 3 experiments are presented as means ± SD. **0.001 < P < 0.01.

In addition, similar results were obtained with a different PG shRNA (Sh2-PG), used to check a potential off-target effect (Supplementary Figure 3). These results were confirmed by an increase in annexin V positive cells after IR in cells expressing PG shRNA (Figure 2B). By western blot, we also analysed the cleavage of poly-(ADP-ribose)-polymerase-1 (PARP-1), a commonly used and well-established apoptotic marker. In both cell line transduced with PG shRNA, we observed a significant increase of cleaved PARP-1, compared to the cells expressing the control shRNA, 3h to 6h after IR indicating an increase of radio-induced apoptosis when PG gene is down-regulated (Figure 2C, 2D). In agreement with these results we also observed after IR an increased activation of the effector caspase 7, known to cleave PARP-1, in both cell lines expressing the PG shRNA compared to the control cells (Figure 3A, 3B). The other effector caspase 3 was undetectable in the cell lines used in this study (data not shown).

In addition to the activation of these effector caspases we also analysed the initiator caspases 8 and 9. Down-regulation of PG gene expression in HCT116 or SW837 cells induced a very high increase in the activation of both caspases under IR, indicating a strong radio-induced apoptosis by targeting PG in CRC cells (Figure 3C, 3D).

**Figure 4:**
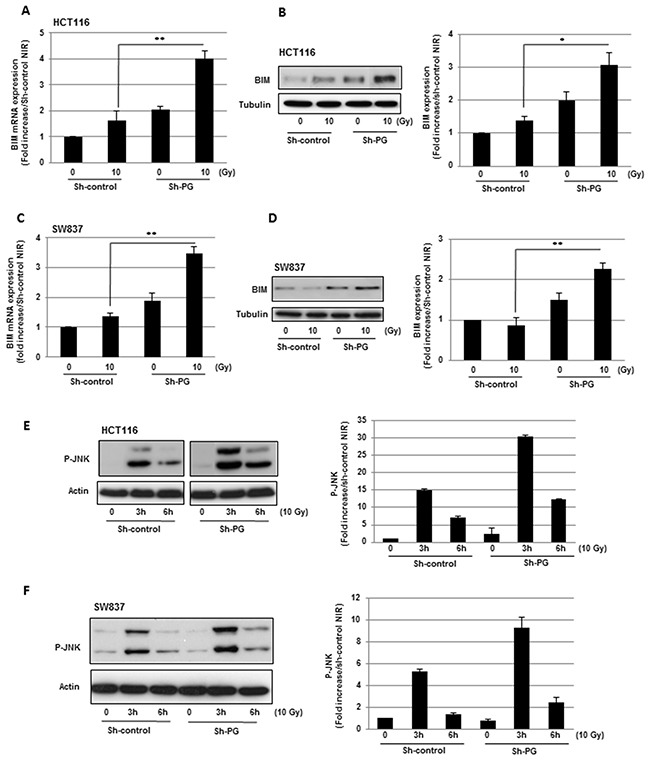
Inhibition of PG gene expression in CRC cells enhances the expression of BIM in response to radiations and increases radio-induced JNK pathway activation BIM expression was measured, 6h post-irradiation, at the mRNA level **(A, C)** using real time PCR and at the protein level **(B, D)** by western blot analysis. Quantifications of 3 experiments are presented as means ± SD.**0.001 < P < 0.01; *0.01 < P < 0.05. **(E, F)** JNK pathway activation was measured by western blot analysis using an anti-phospho-JNK antibody. Quantifications of 2 experiments are presented.

We also analysed the expression of pro-apoptotic mitochondrial proteins, BIM, BAD, BAK and BAX. Only the expression of BIM was significantly increased at the mRNA (Figure 4A, 4C) and protein level (Figure 4B, 4D) after IR in HCT116 and SW837 cells transfected with the PG shRNA compared to the cells expressing the control shRNA. Although it has been previously shown that DNA damages can transcriptionally regulate death receptors, we did not observe any effect of IR, alone or in combination with PG mRNA down-regulation, on the expression of FAS, TNF receptors (TNFR1, TNFR2), TRAIL receptors (R1, R2, R3) as well as FAS Ligand, TNF-α or TRAIL (data not shown).

The Jun N-terminal kinase (JNK) signalling pathway is a key player in radio-induced-apoptosis, in particular by increasing the expression of pro-apoptotic factors. In both cell lines, SW837 and HCT116 expressing the control shRNA, a radiation dose of 10 Gy induced the activation of the JNK pathway with a maximal response 3h after irradiation (Figure 4E, 4F). This effect was increased in cells expressing the PG shRNA.

### Blocking PG gene expression leads to the inhibition of radio-induced pro-survival signalling pathways, AKT and ERK

**Figure 5:**
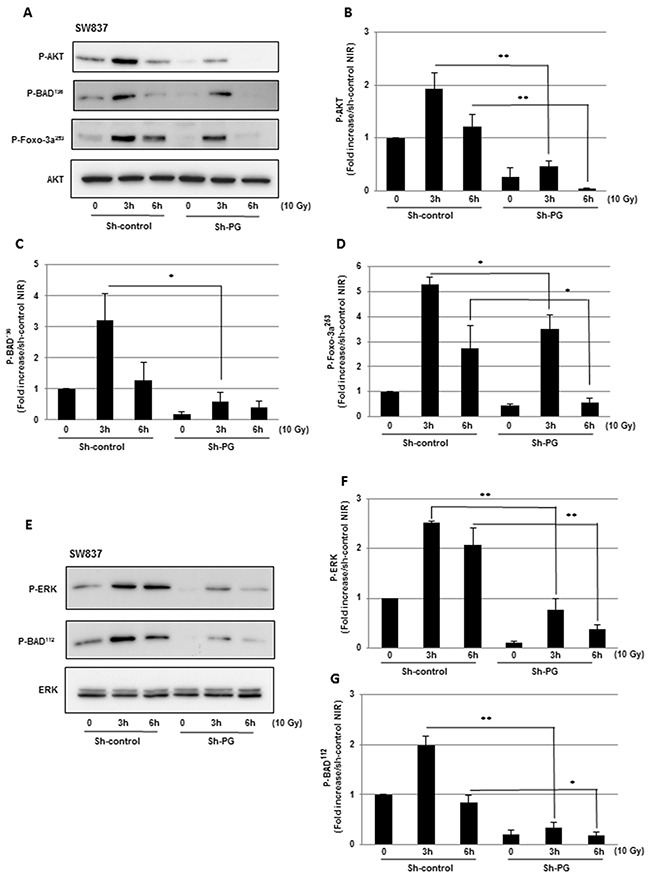
Blocking PG gene expression leads to the inhibition of radio-induced pro-survival signalling pathways, AKT and ERK **(A-D)** Phosphorylation of AKT and its downstream targets BAD and Foxo-3a were measured by western blot analysis using, antibodies targeting phospho-AKT phospho-BAD (Ser 136) or phospho-Foxo-3a (Ser 253). **(E-G)** Phosphorylation of ERK and its downstream target BAD were measured, by western blot analysis using, antibodies targeting phospho-ERK and phospho-BAD (Ser 112). Quantifications of 3 experiments are presented as means ± SD. **0.001 < P < 0.01; *0.01 < P < 0.05.

As mentioned above, exposure to IR induces compensatory activation of survival pathways. Particularly the PI3K-AKT pathway can be induced by IR in different cell types and has been correlated to radioresistance [24]. Therefore to determine if PG contributes to radioresistance through the activation of the PI3K-AKT pathway we analyzed AKT phosphorylation after IR in HCT116 and SW837 cell lines expressing the PG shRNA or a scramble control. In the control HCT116 cells, we observed an increase of AKT phosphorylation in response to IR. However the down-regulation of PG gene by the shRNA did not affect this response (data not shown). In contrast, for the SW837 cell line AKT phosphorylation induced by IR in the control cells was significantly inhibited in cells expressing the PG shRNA (Figure 5A, 5B).

Apoptosis can be regulated by the pro-survival kinase AKT through BAD phosphorylation on the Serine 136 leading to the inhibition of the pro-apoptotic activity of this protein [25]. According to these data, we observed a significant decrease of the radio-induced BAD phosphorylation in the SW837 cells expressing the PG shRNA, (Figure 5A, 5C). Moreover, forkhead-box-O3a (FoxO3a) is a transcription factor mediating apoptosis by regulating the expression of pro-apoptotic factors including BIM. This is also a downstream target of the AKT pathway which inhibits FoxO3a activity by phosphorylation [26, 27]. As expected, FoxO3a phosphorylation after IR, follows AKT phosphorylation in SW837 control cells and is significantly inhibited when PG gene expression is down-regulated (Figure 5A, 5D).

The ERK signalling pathway is another survival pathway known to be activated by IR [24, 25]. This pathway might be activated in resistant tumours cells to diminish radio-induced apoptosis in particular by phosphorylation and inactivation of BAD on Serine 112. In the SW837 control cells, the activation of the ERK pathway was induced in response to radiation. This effect was strongly inhibited in PG-shRNA transfected cell line (Figure 5E, 5F). In accordance with this result, the phosphorylation of BAD on serine 112 observed under IR in SW837 cells expressing control shRNA was also significantly down-regulated in cells when PG gene expression was blocked (Figure 5E, 5G). As previously observed for the AKT pathway, ERK activation was induced by IR in control HCT116 cells, but PG shRNA did not affect this response (data not shown).

### Inhibition of PG gene expression enhances the level of DNA damage induced by radiation

**Figure 6:**
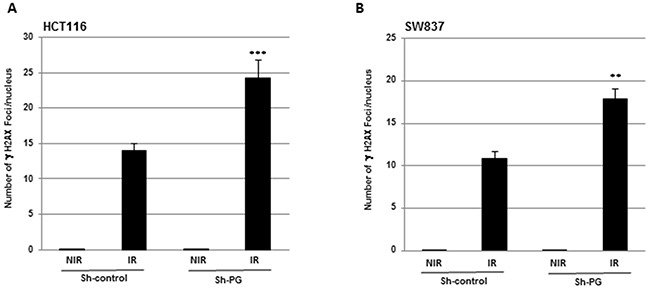
Inhibition of PG gene expression enhances the level of DNA damage induced by radiation HCT116 **(A)** or SW837 **(B)** were grown in chamber slides then irradiated with a single dose of 2 Gy. 24h post-irradiation the number of γH2AX foci was analysed using an AlexaFluor 488 anti-H2AX phosphorylated (Ser139) antibody and a Nikon E400 microscope (X40) with a Sony DXC 950 camera and Visiolab 2000 software. Quantifications of 3 experiments are presented as means ± SD. ***P < 0.001; **0.001 < P < 0.01.

IR is known to induce DNA damages leading to lethal cytotoxicity and low DNA damage repair is an important component of radiation-induced cell death. Therefore we hypothesized that PG downregulation might increase IR-induced DNA damage. To analyse this possibility, we determined whether inhibiting PG gene expression affected IR-induced phosphorylation of H2AX (γ-H2AX), which is a marker of double-strand breaks (DSBs) [28, 29]. In absence of IR we did not observed γ-H2AX staining in cells expressing PG shRNA or the scramble control. In contrast a significant increase in the number of γ-H2AX foci induced by radiations was observed in both cell lines, HCT116 and SW837, transfected with PG shRNA compared to control shRNA (Figure 6A, 6B).

### Down-regulation of PG gene expression radiosensitizes rectal tumours *in vivo*

**Figure 7:**
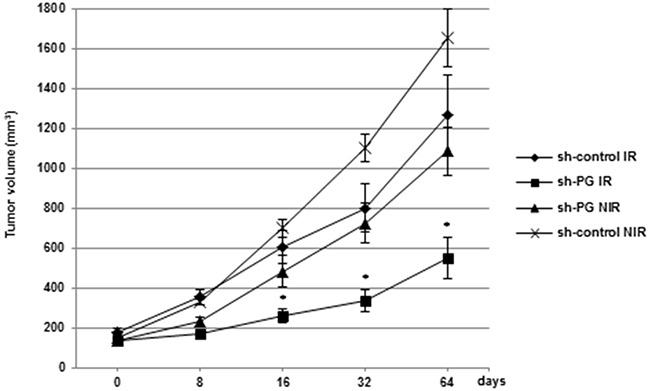
Down-regulation of PG gene expression radiosensitizes rectal tumours *in vivo* SW837 cells stably transfected with a PG shRNA or a scramble control cells were inoculated subcutaneously into athymic nude mice. When the average tumors volume was about 150-200 mm^3^ (day 0), animals were randomized into 4 groups, control-shRNA alone (sh-control NIR), PG-shRNA alone (sh-PG NIR), control-shRNA plus radiation (sh-control IR), PG-shRNA plus radiation (sh-PG IR) and the tumors were locoregionally irradiated with fractionated radiotherapy, 2.5 Gy/fraction every 3 days with a total dose of 10 Gy (IR) or non irradiated (NIR). Tumors sizes were measured twice a week for the duration of the experiment with a digital calliper according to the formula volume = length × (width)^2^ × 0.5. Data are presented as means ± SD, n = 6 animals/group. *0.01 < P < 0.05.

Finally, we evaluated the effect of PG gene down-regulation on the radiosensitization of cancer cells *in vivo*. A tumours growth delay experiment was carried out using the radioresistant rectal cell line, SW837 transfected with PG shRNA or a scramble control. We examined the effect of radiation alone, PG mRNA inhibition alone or the combination of both on the growth of subcutaneous SW837 xenograft into nude mice (Figure 7). From the sixteenth days after randomization and the first irradiation, tumours volume in the group that received the combined treatment (sh-PG IR) was significantly lower than the group with radiations alone (Sh-control IR) or PG mRNA inhibition alone (sh-PG NIR) demonstrating an increased effect with the combined treatment on the radiosensitivity of rectal cancer cells *in vivo*.

## DISCUSSION

A high percentage of advanced rectal tumours are resistant to preoperative chemoradiotherapy which is routinely used in clinic. Therefore targeted therapy against factors involved in radioresistance is an attractive approach to sensitize rectal tumours to the standard preoperative treatment [3]. Consequently, it is extremely important to understand the molecular mechanisms leading to radioresistance and to identify novel molecular targets that can be blocked to increase the sensitivity of rectal tumours to IR. Previous studies showed strong evidence that PG is a growth factor overexpressed in 80-90% of colorectal tumours and plays an important role in carcinogenesis [6–10, 12, 16–19, 22, 30]. However, PG has not been previously associated with resistance to radiotherapy. This prompted us to investigate whether PG overexpression also plays a role in mediating radiation resistance.

We first observed that SW620 and SW837 which are the most radio-resistant cell lines used in this study express the highest levels of PG, suggesting that PG may act as a constitutive radioresistant factor in tumours. However we also observed an increase in PG gene expression after IR indicating that PG may also act as an inducible radioresistant factor. PG gene expression has been previously shown to be activated by the EGF and TGF-beta pathways [31, 32]. Since these two pathways are well known to be activated by radiation [33, 34], they might be involved in the PG gene upregulation observed under radiation in the CRC cell lines. Then to analyse the functional relevance of PG overexpression in mediating radiation resistance, we used a shRNA strategy. Our results showed that PG mRNA down-regulation significantly increased *in vitro* the sensitivity to IR of the different CRC cell lines used in this study. All these cell lines harbor mutations in signalling pathways known to be involved in radioresistance. SW837 and SW620 are mutated for p53, HC116 and DLD1 are mutated in the Pten/PI3K pathway. In addition, all the cell lines are mutated for the Wnt pathway and the K-ras oncogene. Therefore it is important to note that independently of these mutations, we were able to sensitize the CRC cells to IR by targeting PG gene expression. We also showed that the combination of PG gene down-regulation and IR strongly inhibits *in vivo* tumours progression of a highly radioresistant rectal cell line, SW837.

To futher understand the functional mechanism of this radiosensitization following PG gene inhibition, we analyzed radio-induced apoptosis. Although radiation induces mainly the intrinsic apoptotic pathway (mitochondrial), the extrinsic pathway mediated by the death receptors might also contribute to apoptosis induced by irradiation [23]. Here we showed that targeting PG increases radio-induced apoptosis by regulating both pathways. Indeed, following the inhibition of PG gene expression we observed a strong increase of caspases 8 and 9 activation under radiation that converged toward the activation of caspase 7 and the cleavage of PARP.

Several studies have previously established that the JNK pathway plays an important role in radio-induced apoptosis [25]. In addition, crosstalks have been described between the JNK pathway and the caspases cascades. In particular JNK targets include pro-apoptotic genes such as members of the Bcl2 family or death ligands [25]. Similarly to what we observed for the caspases cascades, PG gene inhibition results in a significant increase of radio-induced JNK activation that might explain the increased expression of the Bcl2 family member, BIM. However, JNK can also directly modulate the activity of pro-apototic proteins by phosphorylation. This mechanism cannot be excluded in the cell lines studied here because we observed an increase in the phosphorylation of Bim that followed its expression (data not shown).

IR has been reported to activate survival pathways that play a key role in controlling tumours radio-resistance [24]. Our data also suggest that, in addition to increase radio-induced apoptosis, targeting PG also leads to the inhibition of the PI3K/AKT and ERK pathways induced by IR. This mechanism might also contribute to radiosensitization of colorectal tumours following PG gene inhibition. These results are in accordance with previous papers which showed that inhibitors of these two pathways enhance the radiation responsiveness of colorectal tumors [35, 36]. In addition we showed a crosstalk between the inhibition of these two survival pathways and the activation of the pro-apoptotic pathways regulated by caspases. Indeed down-regulation of the PI3K/AKT pathway and/or the ERK pathway induced by IR following PG gene inhibition led to first, dephosphorylation and activation of the pro-apoptotic protein BAD and second, to dephosphorylation and activation of the transcription factor Foxo3a that might contribute to BIM overexpression.

Several factors involved in NF-kappa B, STAT3, Notch or Wnt pathways have been previously shown to play a role in radioresistance of colorectal tumours [37–41]. Although PG has been previously shown to activate these pathways [18, 42, 43] they do not seem to be involved in PG-mediated radioresistance. Indeed, we did not observed, using PG shRNA, the down-regulation of NF-kappa B or STAT3 activities (measured by western blot; data not shown). In addition the expression (measured by quantitative PCR) of several factors of the Notch or Wnt pathways (Notch 1-3, Jagged 1, DLL1, DLL4, β-catenin) was not decreased in cells expression the PG shRNA compared to the control shRNA (data not shown). However it might be interesting to combine the down-regulation of PG with inhibitors of these pathways to block PG-mediated radioresistance as well as the radioresistance induced by these pathways.

PG has been previously shown to act as an autocrine growth factor, however, the identity of the receptor mediating the PG effects on colonic epithelial cells remains an important point of debate. The data from Jin et al. [44] suggest a role of the CCK2 receptor (CCK2-R), the specific receptor for the mature form of gastrin in the proliferative effects of PG *in vivo*. In contrast, other publications have shown that the CCK2-R is not involved in the PG effects on colon cancer cells [9, 45]. In HCT116 and SW837 cells we did not detect, by quantitative PCR, the expression of the CCK2-R suggesting that in these cell lines the effects of PG are independent of this receptor. The work of Umar et al. [13] suggested that the anti-apoptotic effects of PG may be mediated by the ubiquitous protein Annexin II mainly through the activation of the NF-kappa B pathway in colonic cells. Although our results do not rule out this possibility, we found in the cancer cell lines used in this study that the NF-kappa B pathway is not down-regulated by PG shRNA and is not involved in PG-mediated radioresistance.

Finally, in addition to the induction of apoptosis and a down-regulation of pro-survival pathways by PG targeting, we also demonstrated in this study that PG gene inhibition enhances radiation-induced DNA damage. All these mechanisms might contribute to the radiosensitizing effect of PG shRNA.

In conclusion PG represents an attractive target in CRC since the inhibition of its expression or its activity might decrease cancer cells proliferation, and the radioresistance of rectal tumours.

## MATERIALS AND METHODS

### Cell lines

HCT-116, DLD1, (colon tumours) SW620 (colon tumours, derived from metastasis) and SW837 cells (rectal tumours) were obtained from the American Type Culture Collection, maintained in DMEM supplement with 10% FCS and passaged for fewer than 6 months. These cell lines have been chosen for their different panel of mutations, SW837 and SW620 are mutated for p53 and wild type for the Pten/PI3K pathway, HC116 and DLD1 are mutated in the Pten/PI3K pathway and wild type for p53. All the cell lines are mutated for the Wnt pathway and on the K-ras oncogene.

### Cells irradiation *in vitro*

Cells cultured as specifically described for each methods were exposed to different doses of radiation (2 to 10 Gy) using an Irradiator Gamma-cell Exactor 40 (Nordion, Ottawa, ON, Canada).

### ShRNA transduction, RNA extraction, reverse transcription and real-time PCR

ShRNA directed against PG, obtained from Dr. S. Roche (sh-PG) or Ambion, Applied Biosystems (sh2-PG) were transduced as described previously [19]. Total RNA was isolated by the RNeasy RNA isolation Kit (Qiagen). mRNA expression was determined by real-time PCR, using the ABI-Stepone+(Applied Biosystems). ACTB was used for normalization.

### Survival/clonogenic assay

Cells grown in T25 culture flask or 6 wells plates (1000-1500 cells/wells-flask) for 24h were treated with different doses of gamma rays (2 to 8 Gy). When indicated cells were pre-treated 24h with 1 nM of exogenous PG in absence of serum (Synthetic progastrin, purity> 95% was obtained from NeoMPS polypeptide laboratories, France). After 8-10 days, colonies formed were fixed with 3% paraformaldehyde, stained with crystal violet and those with more than 50 cells were counted. The surviving fraction (SF) was calculated taking into account the plating efficiency (PE).

### Western-blot analysis

Identical levels of proteins were separated by SDS-PAGE and analyzed by western-blot with the indicated antibodies as described previously [20, 21]. Antibodies used for western blot: cleaved-PARP, cleaved-caspase 7, cleaved-caspase 8, cleaved-caspase 9, BIM, phospho-JNK, phospho-AKT, phospho-BAD-ser136, phospho-Foxo-3a-ser253, phospho-ERK, phospho-BAD-ser112, (Cell Signaling), tubulin(SIGMA), and Actin (Millipore). Quantifications were performed with ImageJ software.

### Annexin V staining

Annexin V staining was performed according to the manufacturer protocol (Affimetrix, eBioscience) and cells were analysed 24h post-irradiation by flow cytometry (BD Accuri™ C6 cytometer).

### Sub-G1 analysis

48h post-irradiation, cells were fixed in 70% ice-cold ethanol for 1H at 4 °C. After washing, the cell pellet was resuspended in propidium iodide (PI)-staining buffer (50 μg/ml PI, 10 μg/ml RNAse A) and incubated for 15 min at 37 °C. The DNA content was analyzed by flow cytometry (BD Accuri™ C6 cytometer).

### Immunofluorescent staining of cells

Cells were grown on 12-wells plates containing cover slides. Cells were fixed in 2% paraformaldehyde, permeabilized with 0.1% triton X-100, and incubated with a specific rabbit polyclonal antibody against progastin used for immunofluorescence [22], kindly provided by Pr Shulkes, Melbourne University. The detection was done using a goat anti-rabbit Alexa Fluor 488 antibody (Molecular Probes, Invitrogen, Life Technology). Slides were analyzed with a Nikon E400 microscope (Nikon France) with a Sony DXC 950 camera and Visiolab 2000 software.

### γH2AX staining

Cells were grown in chamber slides for 24h then exposed to a radiation dose of 2 Gy. 24h post-irradiation cells were fixed with 3% paraformaldehyde, permeabilized with 0.1% Triton X-100 and incubated with the AlexaFluor 488 anti-H2AX phosphorylated (Ser139) antibody (BioLegend). Nuclei were counterstained with DAPI. Foci were counted with a Nikon E400 microscope (Nikon France) with a Sony DXC 950 camera and Visiolab 2000 software.

### *In vivo* tumours model

Investigation has been conducted in accordance with the ethical standards according to national and international guidelines and has been approved by the national animalcare committee. 5×10^6^ cells were inoculated subcutaneously into 4 to 5 weeks-old athymic nude mice (Charles River, France). Tumors sizes were measured twice a week with a digital calliper. When the average tumors volume was about 150-200 mm^3^, Animals were randomized into 4 groups (control-shRNA, PG-shRNA, control-shRNA plus radiation, PG-shRNA plus radiation; 6 animals/group). Tumours were locoregionally irradiated with fractionated radiotherapy, 2.5 Gy/fraction every 3 days with a total dose of 10 Gy using gamma rays (Irradiator Gamma-cell Exactor 40, Nordion, Ottawa, ON, Canada).

### Statistical analysis

Means ± SE and Student t Tests were performed using “Excell”. ***P<0.001; **0.001<P<0.01; *0.01<P<0.05; ns P>0.05.

## SUPPLEMENTARY MATERIALS FIGURES



## Abbreviations

CRC: colorectal cancer
PG: progastrin
IR: ionizing radiations
PARP-1: poly-(ADP-ribose)-polymerase-1
DSBs: double-strand breaks
