# Supplementary material for: Targeting progastrin enhances radiosensitization of colorectal cancer cells
Source: Oncotarget. 2017 Apr 20;8(35):58587–600. doi: 10.18632/oncotarget.17274 (PMC5601677; doi:10.18632/oncotarget.17274)
Supplement: Supplementary file 1 [file oncotarget-08-58587-s001.pdf]

Targeting progastrin enhances radiosensitization of colorectal cancer cells

SUPPLEMENTARY MATERIALS

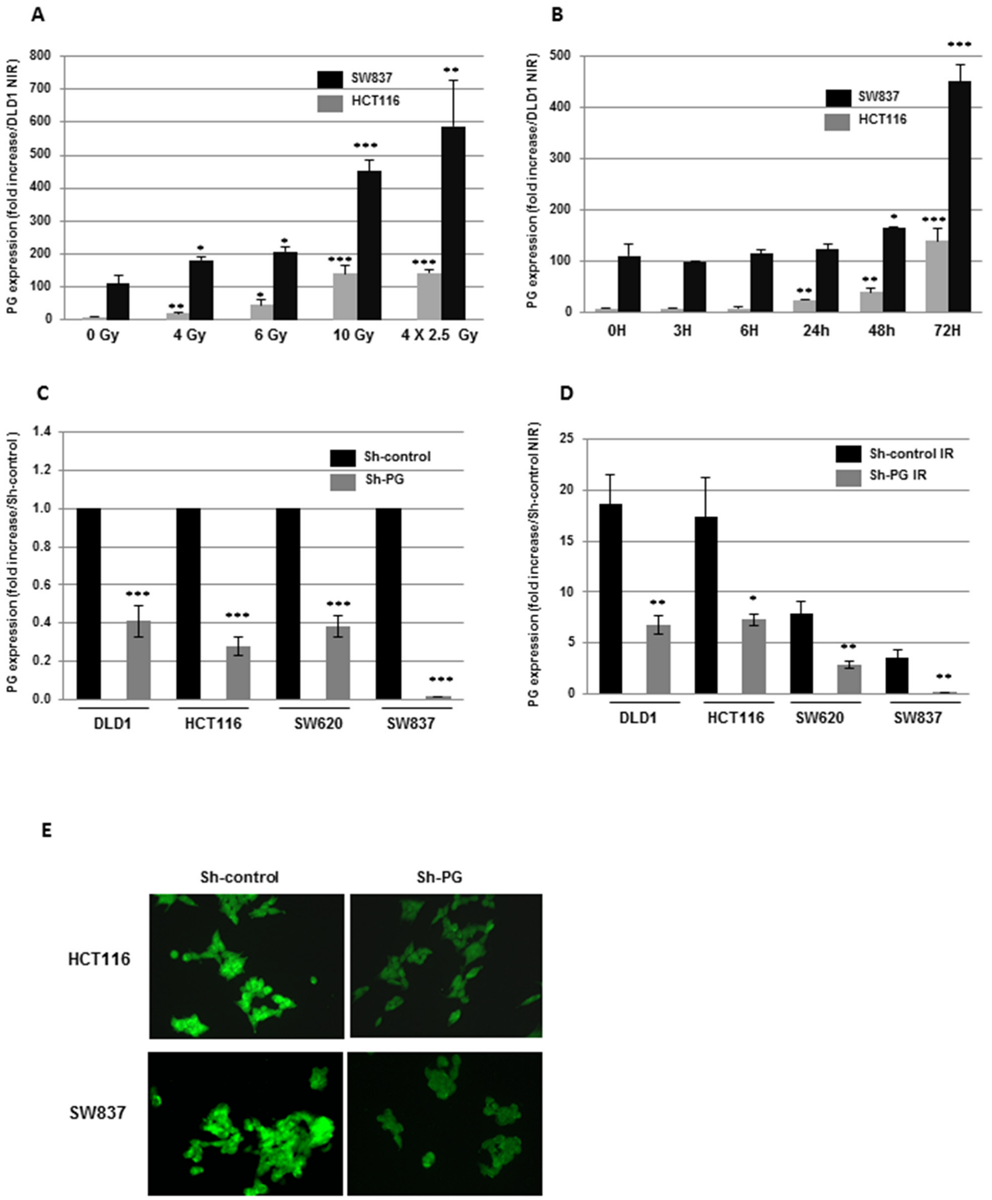

Supplementary Figure 1: PG expression in CRC Cells. (A) HCT116 and SW837 cell lines were exposed to different radiation doses administered as a unique dose (4 to 10 Gy) or by daily multifractions of 4 × 2.5 Gy. PG mRNA expression was quantified 72h post-irradiation. **(B)** HCT116 and SW837 cell lines were exposed to a radiation dose of 10 Gy administered as a unique dose and PG mRNA expression was quantified 3h to 72h post-irradiation as indicated. **(C-E)** Cells were stably transduced with a shRNA directed against the PG gene (Sh-PG) or a scrambled control (Sh-control) as described in "Materials and Methods" and irradiated when indicated with a dose of 10 Gy **(D)**. **(C, D)** PG mRNA expression was measured using real time PCR. Quantifications of 3 experiments are presented as means ± SD. ***P < 0.001; **0.001 < P < 0.01; *0.01 < P < 0.05. **(E)** Cells stably transduced with a shRNA directed against the PG gene (Sh-PG) or a scrambled control (Sh-control) were cultured on cover slides then fixed and stained with a specific PG antibody as described in "Materials and Methods". Representative micrographs (x 40) from 3 independent experiments are shown.

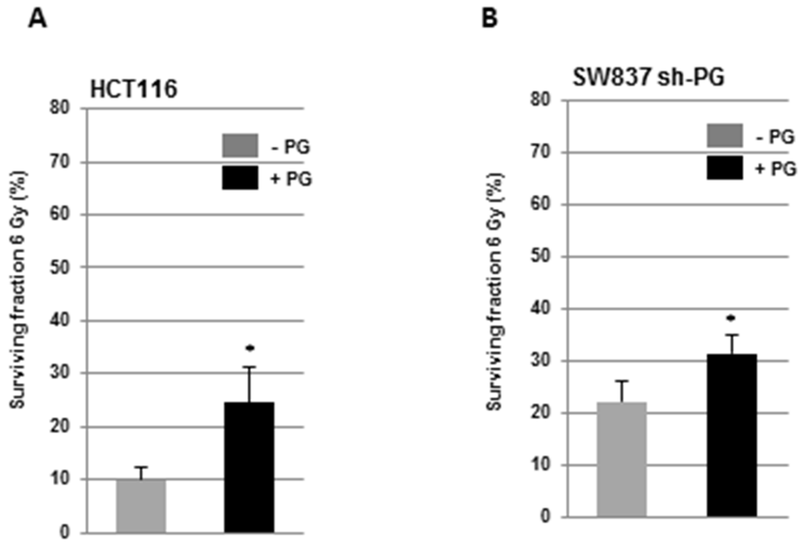

Supplementary Figure 2: PG induces radioresistance of CRC cells *in vitro*. HCT116 cells wild type **(A)** or SW837 stably transduced with a shRNA directed against the PG gene (Sh-PG) **(B)** were pretreated (or not) with 1 nM of PG for 24h in absence of serum before a radiation dose of 6 Gy. Cell survival was measured as described in methods. Quantifications of 3 experiments are presented as means ± SD. *0.01<P<0.05.

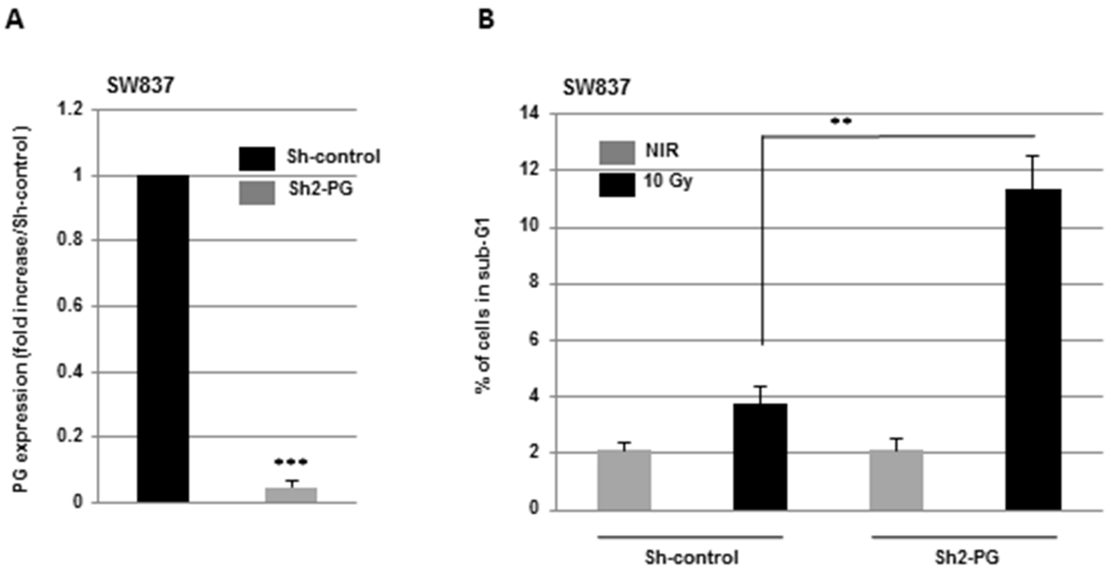

Supplementary Figure 3: PG gene inhibition in SW837 increases radio-induced cell death. **(A)** Cells were stably transduced with a shRNA directed against the PG gene (Sh2-PG) or a scrambled control (Sh-control) as described in "Materials and Methods". PG mRNA expression was measured using real time PCR. **(B)** Propidium iodide staining was performed as described in "Materials and Methods" and the DNA content was analyzed by flow cytometry. Percentages sub-G1 cell population in SW837 are presented. Quantifications of 3 experiments are presented as means ± SD. ***P < 0.001; **0.001 < P < 0.01.
